# The Protection of Ignorance by the U. S. Government

**Published:** 1884-07

**Authors:** 


### THE PROTECTION OF IGNORANCE BY THE U. S. GOVERNMENT.

The majority of the people of this country do not seem to realize that our Government really places a premium on ignorance and a duty on knowledge, by placing import duties upon foreign books. In this regard we find ourselves in full sympathy with those artists that would have foreign works of art admitted free. But while eminently an out-and-out free trader, we cannot but think that there is a striking difference between technical books and most other things that are subjected to the evil influences of our protective tariff.

In subjecting technical books of a foreign origin to an import duty, not a single American author or publisher is benefited in any way. On the contrary, they both suffer from such a duty, and the general public is, in a measure, also the sufferers, as much useful knowledge is thus kept away from authors that would be made available in the writing of books, and many useful books are never written that for want of suggestive material do not emanate from American authors. It is nothing but special legislation and a gross injustice which permits the large colleges, universities, and public libraries to import books and instruments free, thus giving to a favored few that are fortunate enough to be professors of such institutions—oftener possessing more money and social position than brains—advantages which men of far greater genius, living as outside barbarians, cannot obtain. While a student at such an institution, one can have access to its library for any special references, or to its laboratories for any special work; but the moment one graduates, all these are shut off from him, and if unfortunately poor, his scientific tendencies are nipped in the bud, simply for the want of that healthy stimulus which is to be found in foreign scientific literature. Here, as in everything else pertaining to the advancement of science, our Government throws a wet sheet over us to suffocate and smother any attempts at advancement.

Let us take an example. Suppose one of us desires the last report of the Imperial Board of Health of Germany; it costs there 44 marks, or $11. We want to import it, and find the duty is at least one quarter of its cost, besides the expenses; so that in order to benefit nobody but an already surfeited treasury, the unfortunate American seeker after knowledge must pay about three dollars as a penalty for wanting to know something, and being an American citizen.

Surely, while science has no home here—while original research is as foreign to our country in the field of medicine as to an inhabitant of an oasis in the midst of the Great Sahara—it is no great honor or pleasure to be an American citizen, notwithstanding the many civil liberties he may enjoy. Such men would be happier under a despotism like Germany or Russia, where at least the privilege to investigate is fostered, where one finds congenial associates, and where science finds freedom of speech and development when limited to its own fields.

BILLINGS.

